# Short-term Adverse Events After the Third Dose of the BNT162b2 mRNA COVID-19 Vaccine in Adults 60 Years or Older

**DOI:** 10.1001/jamanetworkopen.2022.7657

**Published:** 2022-04-18

**Authors:** Oren Auster, Uriah Finkel, Noa Dagan, Noam Barda, Alon Laufer, Ran D. Balicer, Shay Ben-Shachar

**Affiliations:** 1 Clalit Research Institute, Innovation Division, Clalit Health Services, Tel Aviv, Israel; 2 Software and Information Systems Engineering, Ben Gurion University, Be'er Sheva, Israel; 3 Department of Biomedical Informatics, Harvard Medical School, Boston, Massachusetts; 4 The Ivan and Francesca Berkowitz Family Living Laboratory Collaboration, at Harvard Medical School and Clalit Research Institute, Boston, Massachusetts; 5 Clalit Health Services, Tel Aviv, Israel; 6 School of Public Health, University of Haifa, Haifa, Israel; 7 School of Public Health, Ben Gurion University, Be'er Sheva, Israel; 8 Sackler Faculty of Medicine, Tel Aviv University, Tel Aviv, Israel

## Abstract

This survey study assesses the occurrence of short-term adverse events in adults 60 years or older who received a booster dose of the BNT162B2 mRNA vaccine.

## Introduction

On July 29, 2021, concerns of waning immunity after Pfizer-BioNTech BNT162B2 mRNA vaccination led the Israeli Ministry of Health to start a campaign to administer booster (third) doses to individuals who received their second dose at least 5 months prior.^1,2^ The booster was initially approved for individuals 60 years or older. This survey study assessed the occurrence of adverse effects (AEs) in adults 60 years or older who received a booster dose.

## Methods

This study was conducted among members of Clalit Health Services (CHS), which insures more than half of the Israeli population. Study participants were individuals 60 years or older who received the booster dose of the Pfizer-BioNTech BNT162b2 mRNA COVID-19 vaccine in the first 5 days of the campaign (July 30 to August 3, 2021). The CHS institutional review board approved this study, with a waiver of informed consent because deidentified survey data were used. This study followed the AAPOR reporting guideline for survey studies.

Individuals aged 60 to 79 years were sent a text message with a request to complete an online survey (eTable in the Supplement) regarding AEs. For those 80 years or older, a random sample (41.8%) was contacted by telephone and interviewed.

The survey process comprised 2 sequential surveys conducted 5 to 11 days (August 8-10, 2021) and 20 to 28 days (August 23-26, 2021) after the initiation of the campaign. For participants who responded to both surveys, we considered only the latter response. Individuals’ demographic and clinical characteristics were extracted from their electronic health records.

## Results

Of 82 392 CHS members 60 years or older who received a booster dose during the study period, 66 094 were contacted, with 27 046 (40.9%) responding to the survey. The median age of respondents was 71 years (IQR, 66-75 years); 43.6% of respondents were aged 60 to 69 years; 44.5%, 70 to 79 years; and 12.0%, 80 years or older. The proportion of female respondents was 45.3%, and 49.2% had at least 1 risk factor for severe COVID-19 (Table 1).

**Table 1.** Baseline Characteristics of the Study Population

Characteristic	No. (%)	
	Respondents (N = 27 046)	Nonrespondents (N = 39 020)
Age, median (IQR)	71 (65-75)	71 (66-76)
Age group		
60-69	11 779 (43.6)	16 212 (41.5)
70-79	12 035 (44.5)	18 257 (46.8)
≥80	3232 (12.0)	4551 (11.7)
Sex		
Female	12 258 (45.3)	18 619 (47.7)
Male	14 788 (54.7)	20 401 (52.3)
Population sector		
General Jewish	25 781 (95.3)	36 124 (92.6)
Ultra-Orthodox Jewish	544 (2.0)	1056 (2.7)
Arab	719 (2.7)	1835 (4.7)
Other	2 (<0.1)	5 (<0.1)
Socioeconomic status		
Low	3925 (14.5)	8762 (22.5)
Medium	11 977 (44.3)	17 185 (44.0)
High	10 970 (40.6)	12 618 (32.3)
Missing	174 (0.6)	455 (1.2)
Nursing home resident	320 (1.2)	1331 (3.4)
Risk factors for severe COVID-19^a^		
0	13 740 (50.8)	18 157 (46.5)
1	9100 (33.6)	13 576 (34.8)
2	3092 (11.4)	5264 (13.5)
≥3 or more	1114 (4.1)	2023 (5.2)

^a^ Based on US Centers for Disease Control and Prevention criteria.

Of the respondents, 30.0% reported at least 1 AE, 24.8% reported local reactions, and 16.6% reported systemic reactions. The most common AEs included pain at the injection site (23.5%), fatigue (9.7%), and malaise (7.2%) (Table 2).

**Table 2.** Reports of Adverse Events According to Sex and Age Group

Variable	Respondents, No. (%)					
	Total	Sex		Age, y		
		Female	Male	60-69	70-79	≥80
Any adverse event	8120 (30.0)	4778 (39.0)	3342 (22.6)	3915 (33.2)	3005 (25.0)	1200 (37.0)
Local reaction	6713 (24.8)	4082 (33.3)	2631 (17.8)	3283 (27.9)	2421 (20.1)	1009 (31.1)
Pain at injection site	6346 (23.5)	3853 (31.4)	2493 (16.9)	3109 (26.4)	2288 (19.0)	949 (29.2)
Swelling at injection site	1399 (5.2)	1051 (8.6)	348 (2.4)	688 (5.8)	503 (4.2)	208 (6.4)
Axillary swelling in the injected arm	356 (1.3)	297 (2.4)	59 (0.4)	228 (1.9)	100 (0.8)	28 (0.9)
Other local reaction	1116 (4.1)	773 (6.3)	343 (2.3)	560 (4.8)	437 (3.6)	119 (3.7)
Systemic reaction	4495 (16.6)	2811 (22.9)	1684 (11.4)	2418 (20.5)	1635 (13.6)	442 (13.6)
Fatigue	2634 (9.7)	1727 (14.1)	907 (6.1)	1419 (12.0)	924 (7.7)	291 (9.0)
Malaise	1950 (7.2)	1316 (10.7)	634 (4.3)	1114 (9.5)	660 (5.5)	176 (5.4)
Muscle ache	1506 (5.6)	1041 (8.5)	465 (3.1)	901 (7.6)	500 (4.2)	105 (3.2)
Headache	1326 (4.9)	908 (7.4)	418 (2.8)	771 (6.5)	445 (3.7)	110 (3.4)
Low-grade fever (<38°C)	864 (3.2)	553 (4.5)	311 (2.1)	481 (4.1)	325 (2.7)	58 (1.8)
Joint aches	670 (2.5)	471 (3.8)	199 (1.3)	399 (3.4)	202 (1.7)	69 (2.1)
Nausea	416 (1.5)	325 (2.7)	91 (0.6)	253 (2.1)	115 (1.0)	48 (1.5)
Temperature >38°C	423 (1.6)	290 (2.4)	133 (0.9)	258 (2.2)	135 (1.1)	30 (0.9)
Vomiting or diarrhea	167 (0.6)	123 (1.0)	44 (0.3)	82 (0.7)	60 (0.5)	25 (0.8)
Chest pains	159 (0.6)	103 (0.8)	56 (0.4)	92 (0.8)	52 (0.4)	15 (0.5)
Shortness of breath	110 (0.4)	65 (0.5)	45 (0.3)	56 (0.5)	32 (0.3)	22 (0.7)
Irregular pulse	135 (0.5)	103 (0.8)	32 (0.2)	79 (0.7)	40 (0.3)	16 (0.5)
Disseminated rash	50 (0.2)	29 (0.2)	21 (0.1)	28 (0.2)	16 (0.1)	6 (0.2)
Facial rash	12 (0.0)	11 (0.1)	1 (0.0)	8 (0.1)	2 (0.0)	2 (0.1)
Other systemic reactions	464 (1.7)	271 (2.2)	193 (1.3)	200 (1.7)	178 (1.5)	86 (2.6)
Medical attention for any adverse event	314 (1.2)	187 (1.5)	127 (0.9)	156 (1.3)	104 (0.9)	54 (1.7)
Symptom duration, d						
<1	2350 (8.7)	1265 (10.3)	1085 (7.3)	1119 (9.5)	819 (6.8)	412 (12.7)
1-3	1340 (5.0)	903 (7.4)	437 (3.0)	637 (5.4)	502 (4.2)	201 (6.2)
>3	4330 (16.0)	2567 (20.9)	1763 (11.9)	2115 (18.0)	1639 (13.6)	576 (17.7)
Cannot recall	100 (0.4)	43 (0.4)	57 (0.4)	44 (0.4)	45 (0.4)	11 (0.3)
Adverse events after second dose						
Any reaction	6198 (22.9)	3733 (30.5)	2465 (16.7)	3309 (28.1)	2238 (18.6)	651 (20.0)
No reaction	20 384 (75.4)	8288 (67.6)	12 096 (81.8)	8238 (69.9)	9595 (79.8)	2551 (78.6)
Cannot recall	464 (1.7)	237 (1.9)	227 (1.5)	232 (2.0)	187 (1.6)	45 (1.4)
Third-dose reactions vs second-dose reactions						
Felt better	5064 (18.7)	2266 (18.5)	2798 (18.9)	2537 (21.5)	2171 (18.1)	356 (11.0)
Felt similar	18 345 (67.8)	7818 (63.8)	10 527 (71.2)	7479 (63.5)	8412 (70.0)	2454 (75.6)
Felt worse	3008 (11.1)	1857 (15.1)	1151 (7.8)	1506 (12.8)	1167 (9.7)	335 (10.3)
Cannot recall	629 (2.3)	317 (2.6)	312 (2.1)	257 (2.2)	270 (2.2)	102 (3.1)

Most of the respondents (67.8%) reported that their general feeling after the booster was similar to the feeling after the second dose; 18.7% and 11.1% reported a milder or worse response, respectively. Only 1.2% sought medical attention owing to an AE.

Females were more likely than males to report AEs (39.0% vs 22.6%). The proportion of females who reported systemic reactions was nearly double that of males (22.9% vs 11.4%). Individuals aged 60 to 69 years were more likely to report systemic AEs than were individuals 70 years or older (20.5% vs 13.6%).

## Discussion

We found that AEs after the BNT162b2 mRNA vaccine booster dose were generally mild and usually did not require medical care. The proportion of self-reported AEs that occurred in our study was similar or lower than that after the administration of the second vaccine dose in several previous studies.^3,4^ A study by Menni et al^4^ found a similar proportion of systemic reactions among older individuals after the second vaccine dose as after the third dose in our study (16.4% vs 16.6%).

The proportion of female respondents who reported systemic AEs was greater than the proportion of male respondents, with higher proportions among participants in the younger age group (60-69 years) than in the older age groups. Similar results were reported in previous studies after administration of the second vaccine dose.^3,4,5^ A limitation of our study was the different survey methods in different age groups, which might have resulted in differences in reported proportions of AEs.

## Notes

Supplement. **eTable.** Survey Questionnaire
