# Supplementary material for: Short-term Adverse Events After the Third Dose of the BNT162b2 mRNA COVID-19 Vaccine in Adults 60 Years or Older
Source: JAMA Netw Open. 2022 Apr 18;5(4):e227657. doi: 10.1001/jamanetworkopen.2022.7657 (PMC9016488; doi:10.1001/jamanetworkopen.2022.7657)
Supplement: Supplement. — eTable. Survey Questionnaire [file jamanetwopen-e227657-s001.pdf]

Supplemental Online Content

Auster O, Finkel U, Dagan N, et al. Short-term adverse events after the third dose of the BNT162b2 mRNA COVID-19 vaccine in adults 60 years or older. *JAMA Netw Open*. 2022;5(4):e227657. doi:10.1001/jamanetworkopen.2022.7657

eTable. Survey Questionnaire

This supplemental material has been provided by the authors to give readers additional information about their work.

© 2022 Auster O et al. *JAMA Network Open.*

eTable – Survey questionnaire

#	Question	Conditions†
Q1	Did you receive the third dose of the COVID-19 vaccine in the last two weeks?<br><br>*(Yes / No)*	
Q2	Did you experience any adverse events following the third vaccine dose?<br><br>*(Yes / No)*	
Q3	Did you have any local reaction in the arm in which the vaccine was injected?<br><br>*(Yes / No)*	Q2 = "yes"
Q4	Which of the following local reactions did you have in the arm of the injection?<br><br>*(Multiple choice)*<br><br>- *Pain at the injection site*<br>- *Swelling at the injection site*<br>- *Swelling of the armpit, near the injection site*<br>- *Other reaction*	Q2, Q3 = "yes"
Q5	Did you have any local reactions in the rest of the body following the injection of the vaccine?<br><br>*(Yes / No)*	Q2 = "yes"
Q6	Which of the following adverse events in the rest of the body did you experience?<br><br>*[Multiple choice]*<br><br>- *Fatigue*<br>- *Headache*<br>- *Muscle aches*<br>- *Fever up to 38 degrees [Celsius]*<br>- *Fever above 38 degrees [Celsius]*<br>- *Nausea*<br>- *Vomiting or diarrhea*<br>- *Joint aches*<br>- *Generally feeling bad [=malaise]*<br>- *Disseminated rash*<br>- *Rash on the face*<br>- *Chest pains*<br>- *Irregular pulse*<br>- *Difficulty breathing*<br>- *Other reaction*	Q2, Q3 = "yes"
Q7	For how many days following the administration of the third vaccine dose did the adverse reactions last?<br><br>- *Up to one day*<br>- *One to two days*<br>- *Two to three days*<br>- *Over three days*<br>- *Cannot recall*	Q2 = "yes"

© 2022 Auster O et al. *JAMA Network Open.*

Q8	Did you seek medical attention for one or more of the adverse events?<br><br><u>*First survey*</u>*:*<br><br>*Yes / No*<br><br><u>*Second survey*</u>*:*<br><br>- *Yes, at the hospital*<br>- *Yes, community doctor*<br>- *No*	Q2 = "yes"
Q9	Several months ago, you received the second dose of the vaccine – did you experience any adverse events following the administration of the second vaccine?<br><br>- *Yes*<br>- *No*<br>- *Cannot recall*	
Q10	How would you summarize your feeling in the days after receiving the third vaccine dose, in comparison to the days after receiving the second vaccine dose?<br><br>- *Feel better now*<br>- *Feel similar*<br>- *Feel worse now*<br>- *Cannot recall*	

This survey was translated from Hebrew.

† Only participants who met the condition were asked the relevant question, i.e., only participants who answered "yes" in Q2 were asked Q3.

© 2022 Auster O et al. *JAMA Network Open.*
